# DNA Double-Strand Break Repair: All Roads Lead to HeterochROMAtin Marks

**DOI:** 10.3389/fgene.2021.730696

**Published:** 2021-09-01

**Authors:** Pierre Caron, Enrico Pobega, Sophie E. Polo

**Affiliations:** Epigenetics and Cell Fate Centre, CNRS, University of Paris, Paris, France

**Keywords:** chromatin mobility, chromatin remodeling factors, DNA double-strand break repair pathway choice, heterochromatin, histone variants, histone modifications

## Abstract

In response to DNA double-strand breaks (DSBs), chromatin modifications orchestrate DNA repair pathways thus safeguarding genome integrity. Recent studies have uncovered a key role for heterochromatin marks and associated factors in shaping DSB repair within the nucleus. In this review, we present our current knowledge of the interplay between heterochromatin marks and DSB repair. We discuss the impact of heterochromatin features, either pre-existing in heterochromatin domains or *de novo* established in euchromatin, on DSB repair pathway choice. We emphasize how heterochromatin decompaction and mobility further support DSB repair, focusing on recent mechanistic insights into these processes. Finally, we speculate about potential molecular players involved in the maintenance or the erasure of heterochromatin marks following DSB repair, and their implications for restoring epigenome function and integrity.

## Introduction: Diversity and Functional Importance of Heterochromatin Marks

The organization of the genome into chromatin in the nuclear space serves to precisely orchestrate cellular functions by controlling gene expression. While the euchromatin compartment is generally accessible and associated with active gene transcription, heterochromatin is more condensed and mostly transcriptionally silent (Allshire and Madhani, 2018). Beyond this general definition, heterochromatin domains are actually quite diverse, in localization, regulation, and function. Constitutive heterochromatin is highly conserved between different cell types, rich in repeated sequences, and plays critical roles in the maintenance of chromosomal architecture and stability (Janssen et al., 2018; Penagos-Puig and Furlan-Magaril, 2020). The bulk of constitutive heterochromatin forms at pericentromeric regions, which are involved in the control of chromosomal segregation (Saksouk et al., 2015). Likewise, telomeres adopt a specific constitutive heterochromatin structure that serves to shield chromosomal ends from aberrant DNA repair, thus protecting chromosome integrity (de Lange, 2002; Allshire and Madhani, 2018; Lim and Cech, 2021). Facultative heterochromatin, in contrast, is developmentally regulated and varies across cell types. Its main function is to silence gene regions that should not be expressed in a specific developmental or somatic context (Trojer and Reinberg, 2007). The inactive X chromosome (Xi) is a typical example of facultative heterochromatin, which is established early during female mammalian development for the dosage compensation of X-linked genes (Galupa and Heard, 2018). Heterochromatin also forms at the nuclear periphery through interactions with the nuclear lamina leading to lamina-associated domains (LADs), which play an important role in chromosome organization and gene repression (van Steensel and Belmont, 2017).

The establishment and maintenance of the silent state in heterochromatin domains involve DNA methylation and repressive histone post-translational modifications (PTMs). While constitutive and facultative heterochromatin are both enriched in DNA methylation, they show specific histone PTMs. Constitutive heterochromatin is enriched in H3K9me2/3, which is bound by heterochromatin protein 1 (HP1), a factor that plays a crucial role in heterochromatin assembly (Saksouk et al., 2015). Facultative heterochromatin instead shows an enrichment of H3K27me3 and H2AK119ub (Galupa and Heard, 2018), and the facultative heterochromatin mark H3K27me3 is also enriched in LADs (van Steensel and Belmont, 2017). In addition to DNA and histone modifications, heterochromatin domains incorporate specific histone variants (Martire and Banaszynski, 2020), such as centromere protein A (CENP-A) at centromeres and macroH2A1 in the Xi, and associate with architectural factors that determine the three-dimensional chromatin structure. In this review, DNA and histone modifications, histone readers, histone variants, and architectural factors enriched in heterochromatin domains are collectively referred to as heterochromatin marks or features. Remodeling factors (Clapier et al., 2017) provide another layer of regulation of heterochromatin accessibility by affecting nucleosome positioning. All these factors come into play to shape heterochromatin domains and mediate their function (Allshire and Madhani, 2018).

One of the major functions of heterochromatin is to ensure a tight control of transcriptional states, which is key for maintaining genome integrity and cell fate (Janssen et al., 2018). Nevertheless, heterochromatin domains also represent challenging environments for DNA metabolic activities, including DNA replication and DNA damage repair (Fortuny and Polo, 2018). Indeed, these domains are late replicating, highly compact, and often encompass repetitive sequences, which contributes to replication stress and fuels genome instability. Heterochromatin repeats are also prone to instability through ectopic recombination leading to deletions or translocations. Moreover, the highly compacted state and the low transcriptional activity in heterochromatin domains impede several repair pathways (Fortuny and Polo, 2018). These obstacles can be circumvented by alterations of the heterochromatin structure during the repair process.

Among the many types of DNA lesions, highly cytotoxic DNA double-strand breaks (DSBs) are repaired by multiple pathways with different levels of fidelity. Non-homologous end joining (NHEJ) is predominant and proceeds by direct ligation of DNA ends, while homologous recombination (HR) requires an initial resection of the DNA ends followed by recombination with a homologous template, usually the sister chromatid, which restricts HR to the S and G2 phases of the cell cycle (Chen et al., 2018; Zhao et al., 2020). Single-strand annealing (SSA) is based on homology on the same DNA strand and repairs DSBs between repeated sequences, leading to large deletions (Bhargava et al., 2016). DNA double-strand break repair by microhomology-mediated end joining (MMEJ) is also highly mutagenic, as it relies on short microhomology sequences that are exposed after end resection, and always generates small indels (Sallmyr and Tomkinson, 2018). The choice between several DSB repair pathways with different degrees of mutagenicity is thus decisive for the maintenance of genomic stability and is subject to complex regulatory mechanisms (Scully et al., 2019), including at the chromatin level. Recent studies have uncovered the key role of heterochromatin marks in dictating DSB repair pathway choice. DNA double-strand break repair, in turn, involves alterations in heterochromatin organization and heterochromatin marks, which need to be reverted to preserve epigenome integrity.

Here, by focusing on recent discoveries in the field, we provide an overview of our current knowledge of the interplay between heterochromatin marks and DSB repair and discuss potential mechanisms that preserve the integrity of heterochromatin domains.

## Heterochromatin Features Direct DSB Repair Pathway Choice

Heterochromatin marks and associated factors not only play critical roles in transcriptional silencing but also contribute to regulate DSB repair pathway choice, in part by controlling the recruitment of DSB repair factors. This regulation has been observed both in heterochromatin domains where heterochromatin marks are present before damage infliction and in euchromatin domains where DSBs trigger the deposition of specific heterochromatin marks. In this section, we discuss recent studies that provided new mechanistic insights into the regulation exerted by heterochromatin marks on DSB repair.

### Role of Heterochromatin-Specific Histone Modifications in DSB Repair Pathway Choice

Histone PTMs constitute an important layer of epigenomic information with a broad impact on chromatin organization and function; some of these marks define heterochromatin domains and have been shown to regulate DSB repair responses.

For instance, a well-known PTM enriched in constitutive heterochromatin is H3K9me3. As previously described, an increase of this mark was observed at break sites in mammalian cells, both in heterochromatin and in euchromatin regions (Ayrapetov et al., 2014; Tsouroula et al., 2016; Natale et al., 2017), and several players in the H3K9me3 pathway – writers (SUV39H1/2, SETDB1) and readers (HP1, TIP60) – were shown to promote DSB repair by HR (Sun et al., 2009; Baldeyron et al., 2011; Soria and Almouzni, 2013; Tang et al., 2013; Alagoz et al., 2015; Jacquet et al., 2016). In line with these studies, the H3K9 methyltransferase SET domain bifurcated histone lysine methyltransferase 1 (SETDB1) was also shown to regulate alternative lengthening of telomeres (ALT) in mouse cells by creating an H3K9me3-rich heterochromatin environment that facilitates recombination (Gauchier et al., 2019).

Recent studies have elucidated the mechanism underlying the deposition of H3K9me3 around DSBs and described new interactions between this histone modification and the regulation of DSB repair. It was shown that H3K9me3 actually depends on another damage-induced PTM on histone H4. The DSB sensor complex MRE11-RAD50-NBS1 indeed recruits UFM1-specific ligase 1, leading to the conjugation of a ubiquitin-like protein to histone H4 lysine 31, a process known as ufmylation. This histone PTM is bound by the serine/threonine-protein kinase 38 (STK38), which in turn recruits the H3K9 methyltransferase suppressor of variegation 3–9 homolog 1 (SUV39H1) leading to the trimethylation of H3K9 around DSBs (Qin et al., 2019, 2020). The local increase of H3K9me3 at DSB sites seems to be crucial for HR as shown in human cancers with elevated levels of oncometabolites that inhibit the lysine demethylase KDM4B. This causes an aberrant constitutive hypermethylation of H3K9 instead of a local increase at break sites, which diverts TIP60 away from the DSBs, thereby impairing HR activation (Sulkowski et al., 2020).

The local increase of H3K9me3 points toward a heterochromatinization phenomenon that may be necessary for HR repair. However, one also has to consider how the DSB repair machinery handles breaks in heterochromatin regions that are already decorated with this mark. Indeed, HR could lead to mutagenic recombination in heterochromatin compartments due to their highly repetitive nature. Several strategies actually serve to prevent HR in H3K9me3-containing heterochromatin domains. In mouse cells for instance, resection of the DNA ends leads to their relocalization to the periphery of pericentromeric heterochromatin, where recombination takes place (Tsouroula et al., 2016). In *Drosophila melanogaster* in contrast, DSBs in pericentromeric heterochromatin trigger the recruitment of *Drosophila* lysine demethylase 4a, which demethylates H3K9me3 and H3K56me3, another conserved pericentromeric heterochromatin mark (Jack et al., 2013). This demethylase channels repair to NHEJ by inhibiting the recruitment of early HR factors to heterochromatic DSBs (Janssen et al., 2019).

In addition to H3K9me3, H3K27me3 also decorates heterochromatin regions, such as those associated with the lamina, and DSBs in these H3K27me3-enriched regions show increased repair by MMEJ (Lemaître et al., 2014; Schep et al., 2021). Interestingly, chemical inhibition of H3K27 and not of H3K9 methyltransferases shifted the MMEJ/NHEJ balance toward NHEJ (Schep et al., 2021), arguing that the H3K27me3 heterochromatin mark either stimulates MMEJ or inhibits NHEJ. However, the underlying molecular mechanisms still need to be elucidated and the impact of H3K27me3 on HR is unknown. Similar to H3K9me3, H3K27me3 was also found increased at DSBs in some studies but with conflicting results in other studies (reviewed in Ferrand et al., 2020), so further work is needed to clarify the status of this mark at DSBs in and outside heterochromatin.

Besides H3K9me3 and H3K27me3, other histone modifications play a role in DSB repair regulation in heterochromatin domains. For instance, several H3K36me2-specific histone methyltransferases, including multiple myeloma SET domain-containing protein (MMSET), promote NHEJ at deprotected telomeres in mouse cells. Interestingly, the involvement of H3K36me2 seems to occur downstream of DSB recognition and repair pathway choice (de Krijger et al., 2020).

While the mechanisms through which H3K9me3 impacts DSB repair are now quite well characterized, how other histone marks, like H3K27me3 and H3K36me2, influence this process is still unknown. Further studies are necessary to determine whether those marks modulate the recruitment of specific DSB repair factors to chromatin. In addition, it will be interesting to investigate whether histone PTMs also underlie the differential regulation of HR between centromeric and pericentromeric heterochromatin observed in mouse cells (Tsouroula et al., 2016). Homologous recombination of centromeric DSBs is indeed licensed in G1, in addition to S/G2, which could rely on histone marks decorating centromeres, such as H3K4me2, H3K36me2, and H3 acetylation (Chan and Wong, 2012).

### Role of Heterochromatin-Specific Histone Variants in DSB Repair Pathway Choice

Besides histone modifications, another layer of chromatin regulation builds upon the incorporation of histone variants (Martire and Banaszynski, 2020), some of which are enriched in heterochromatin and regulate DSB repair. Such regulation occurs in already histone variant-enriched heterochromatin domains and also upon the accumulation of these variants at euchromatic DSBs.

The histone variant MacroH2A1, for instance, is enriched in facultative heterochromatin domains on autosomes and on the inactive X chromosome (Costanzi and Pehrson, 1998; Changolkar and Pehrson, 2006; Gamble et al., 2010). Remarkably, the macroH2A1 gene expresses two splicing isoforms: macroH2A1.1 and macroH2A1.2, which exhibit antagonistic properties in the regulation of DSB repair pathway choice in mammalian cells. MacroH2A1.2 accumulates at DSBs in an ataxia telangiectasia mutated (ATM)-dependent manner and stimulates DSB repair by HR by promoting the recruitment of breast cancer type 1 susceptibility protein (BRCA1; Khurana et al., 2014). Similarly, macroH2A1.2 deposition at sites of replication stress by the histone chaperone facilitates chromatin transcription (FACT) forms a chromatin environment amenable for BRCA1 recruitment (Kim et al., 2017). In human cells lacking the chromatin remodeler Alpha thalassemia/mental retardation syndrome X-linked (ATRX), macroH2A1.2 is also highly enriched at telomeres and contributes to ALT, a HR-mediated process (Kim et al., 2019). Mechanistically, macroH2A1.2 collaborates with the histone demethylase KDM5A to promote both DSB repair by HR and transcriptional silencing at breaks (Kumbhar et al., 2021). MacroH2A1.1 in contrast supports MMEJ, a mutagenic DSB repair pathway (Sebastian et al., 2020). The preferential interaction of macroH2A1.1 with MMEJ repair factors, including poly(ADP-ribose) polymerase 1 (PARP1), is likely linked to the ability of this isoform to bind ADP-ribose, a property that is not shared with macroH2A1.2 (Sebastian et al., 2020). MacroH2A1.2-deficient cells display X-chromosome instability due to defective HR and enhanced MMEJ. Interestingly, loss of macroH2A1.1 rescues the X-chromosome instability observed in macroH2A1.2-deficient cells (Sebastian et al., 2020). This nicely illustrates how histone variants exert antagonistic control on DSB repair pathway choice and genome integrity in facultative heterochromatin.

Another histone variant that may regulate DSB repair is CENP-A, which defines centromeric heterochromatin. There is conflicting evidence regarding CENP-A accumulation post DSBs (Zeitlin et al., 2009; Ambartsumyan et al., 2010; Helfricht et al., 2013), and the link between CENP-A and DSB repair has not yet been explored. It will be interesting to investigate whether CENP-A can contribute to licensing HR in G1 at centromeric DSBs (Tsouroula et al., 2016). Furthermore, the CENP-A chaperone HJURP may have functional connections to DSB repair (Kato et al., 2007).

### The Role of Heterochromatin-Associated Factors in DSB Repair Pathway Choice

In addition to histone variants and modifications, several heterochromatin-associated factors play a central role in DSB repair, including histone readers, architectural factors, and chromatin remodelers.

Chromatin remodelers were shown to regulate chromatin relaxation at heterochromatic DSBs in mammalian cells. In this respect, imitation switch (ISWI)-class and chromodomain-helicase-DNA binding (CHD)-class chromatin remodelers play antagonistic roles. CHD3 promotes heterochromatin compaction, but is released from chromatin following ATM activation, while ACF1 and SNF2H (ISWI class) are recruited to the damage site and lead to heterochromatin decompaction, which allows Artemis-dependent NHEJ (Klement et al., 2014). A recent study put forward the involvement of another chromatin remodeler in stimulating DSB repair by HR in heterochromatin. The human chromatin remodeler lymphoid-specific helicase (HELLS), through its ATPase activity, indeed promotes HR of heterochromatic DSBs in G2 cells exposed to ionizing radiation by facilitating end resection through CTBP-interacting protein (CtIP) recruitment (Kollárovič et al., 2020). Whether the function of HELLS in HR repair of heterochromatic breaks is linked to its ability to promote macroH2A1.2 deposition (Ni et al., 2020; Xu et al., 2021) is an intriguing possibility that deserves further investigation. The involvement of multiple remodelers, some of which having opposing activities, likely allows a fine-tuning of heterochromatin compaction during DSB repair, with dynamic changes over time after DSB induction.

Among readers of heterochromatin-specific modifications, HP1 is recruited to DSBs arising in euchromatin and heterochromatin domains, and a major regulator of DSB repair, with HP1 isoforms having different effects on DSB repair pathways: HP1α and β stimulate HR at the resection step, while HP1γ inhibits this pathway (Baldeyron et al., 2011; Soria and Almouzni, 2013). In line with these findings, HP1γ depletion, but not that of HP1α and β, negatively impacts Ku80 recruitment to heterochromatic DSBs in mouse cells (Tsouroula et al., 2016), suggesting that the HP1γ isoform may play a role in NHEJ. A possible mechanism through which some HP1 isoforms channel DSB repair toward HR might rely on the direct binding of HP1 to BRCA1-associated RING domain 1 (BARD1) in response to DSBs, which promotes retention of the BARD1-BRCA1 complex stimulating CtIP-dependent resection (Wu et al., 2015).

Finally, the heterochromatin-enriched architectural factor structural maintenance of chromosomes flexible hinge domain containing 1 (SMCHD1) contributes both to transcriptional silencing and to DSB repair. It is still unknown if both functions of SMCHD1 are mechanistically connected. This protein indeed plays a crucial role in mammalian X chromosome inactivation (Blewitt et al., 2008) and is involved in the silencing of specific autosomal genes (Gendrel et al., 2013; Mould et al., 2013). In addition, SMCHD1 is recruited to DNA damage foci (Coker and Brockdorff, 2014; Tang et al., 2014) and is highly enriched on deprotected telomeres in human cells (Vancevska et al., 2020), pointing to a role in the DSB response. SMCHD1 actually contributes to DSB repair pathway choice by promoting NHEJ while inhibiting HR, as shown using cell reporter systems (Tang et al., 2014). Consistent with this, SMCHD1 stimulates 53BP1 foci formation and impairs BRCA1 foci formation following cell treatment with the radiomimetic drug zeocin (Tang et al., 2014). SMCHD1 also promotes the fusion of unprotected telomeres, which relies on NHEJ; however, the function of SMCHD1 seems to be upstream of DSB repair at telomeres through the stimulation of ATM-dependent damage signaling (Vancevska et al., 2020).

**Figure 1:**
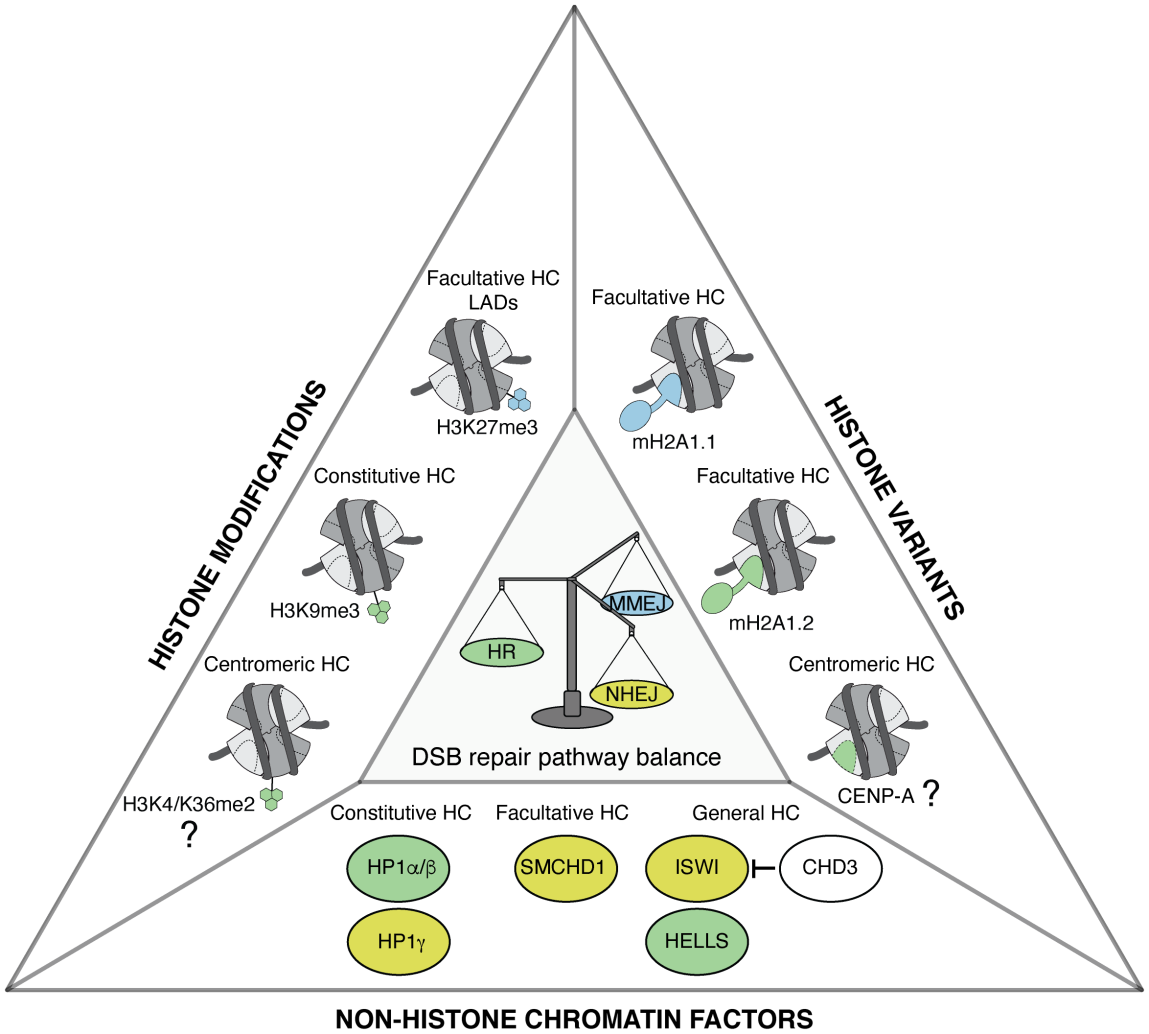
Heterochromatin features govern DNA double-strand break (DSB) repair pathway choice. Representation of heterochromatic (HC) histone modifications, histone variants, and non-histone chromatin factors that modulate DSB repair pathway choice in mammalian cells. Features that favor homologous recombination (HR), non-homologous end joining (NHEJ), and microhomology-mediated end joining (MMEJ) are shown in green, yellow, and blue, respectively. The types of heterochromatin enriched in these features are indicated when known. The contribution of centromeric histone variant and modifications to promoting HR in G1 is still to be determined, as indicated by the question marks.

Together, these studies illustrate that several heterochromatin marks, including histone trimethylation, histone variants, and non-histone proteins, regulate DSB repair pathway choice (Figure 1). Interestingly, some marks with opposing activities on DSB repair are enriched in the same heterochromatin domain, as observed for macroH2A1.1, 1.2, SMCHD1, and H3K27me3 on the Xi. This might suggest an interplay between heterochromatin marks, which could allow a fine regulation of DSB repair pathways. Notably, in addition to HR, NHEJ, and MMEJ, DSB repair by SSA also operates in heterochromatin, in particular when HR is compromised (Janssen et al., 2016; Tsouroula et al., 2016), but whether heterochromatin marks stimulate SSA is still unknown. Further studies are necessary to clarify the mechanisms through which heterochromatin marks modulate DSB repair and to assess the combinatorial effects of these marks.

## Alteration and Maintenance of Heterochromatin Features in Response to DSBs

While the choice of repair pathway is influenced by the chromatin context, DSB repair itself leads to changes in heterochromatin organization, which can have a profound impact on its function and thus on the maintenance of genomic integrity.

### Heterochromatin Decompaction and Mobility of Heterochromatic Breaks

Heterochromatin is a highly compacted nucleoprotein structure that can be seen as an obstacle for the detection of DNA lesions and their repair. However, in mammals and *D. melanogaster*, it was observed that DSB repair kinetics were comparable between heterochromatin and euchromatin (Goodarzi et al., 2008; Janssen et al., 2016). This is achieved, at least in part, through the decompaction of heterochromatin, which facilitates DSB signaling and repair. Mechanistically, heterochromatin decompaction is regulated by chromatin remodelers, as discussed above, and by the ATM kinase, which phosphorylates KRAB-associated protein 1 (KAP-1) thus triggering its eviction from chromatin (Ziv et al., 2006; Goodarzi et al., 2008). Heterochromatin decompaction has been observed in several heterochromatin compartments in response to DSBs induced by ionizing radiation or by site-specific nucleases, including the Xi compartment in female mammalian cells (Müller et al., 2013) and pericentromeric heterochromatin domains in *Drosophila* and mouse cells (Chiolo et al., 2011; Tsouroula et al., 2016). Of note, the expansion of pericentromeric regions is not specific to the DSB response as it is also observed upon UV damage detection by DNA damage-binding protein 2 (DDB2), which triggers the eviction of linker histone H1 from chromatin (Fortuny et al., 2021). H1 eviction is also reported in response to DSBs (Strickfaden et al., 2016; Clouaire et al., 2018; Li et al., 2018), but whether linker histone eviction participates to heterochromatin decompaction post DSBs is not yet known.

Mechanistically, chromatin decompaction is accompanied by DSB repositioning outside of pericentromeric heterochromatin domains, favoring repair completion through HR, as shown in both *Drosophila* and mouse cells (Chiolo et al., 2011; Tsouroula et al., 2016; Amaral et al., 2017). In both systems, the resection of the broken ends occurs within pericentromeric heterochromatin and triggers their migration to the periphery of these domains in mouse cells, and to the nuclear periphery in *Drosophila* cells, where RAD51-mediated recombination takes place (Chiolo et al., 2011; Tsouroula et al., 2016). A similar process may occur in response to DSBs in the Xi. Indeed, while 53BP1 is found both within and outside of the Xi, phosphorylated RPA localizes at the Xi periphery, indicating a possible relocalization of the breaks undergoing resection, to be repaired by HR (Müller et al., 2013). Functionally, such relocalization of breaks outside heterochromatin domains is thought to prevent aberrant ectopic recombination between repeated sequences.

**Figure 2:**
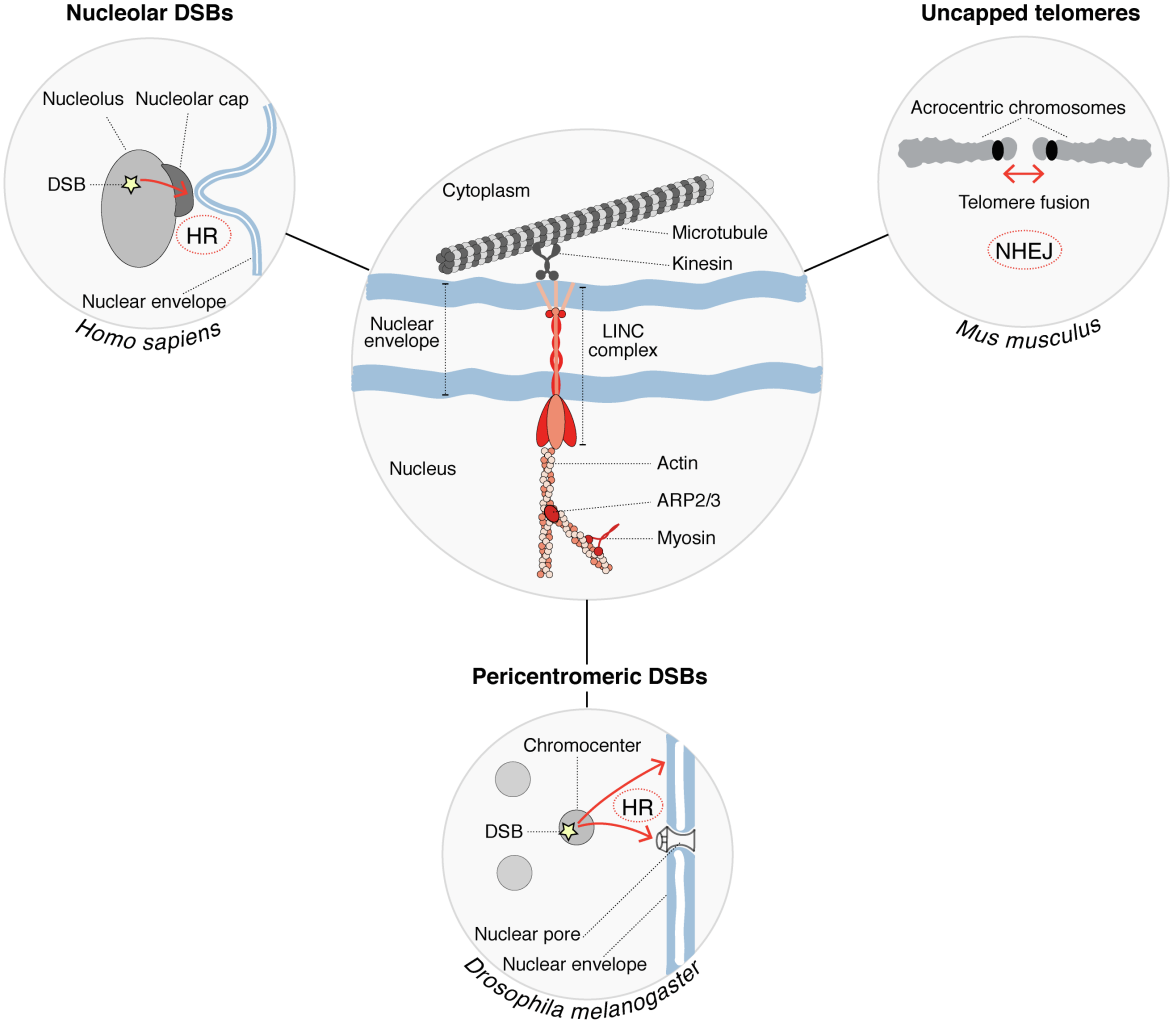
Cytoskeleton factors regulate the mobility of damaged heterochromatin to support DSB repair. Nucleoskeleton factors and molecular motors (shown in red in the center circle) are involved in the mobility of heterochromatic DSBs (DSB mobility is represented by red arrows in the peripheral circles). Chromatin mobility is further supported by microtubules, which provide mechanical forces. This process is conserved in several species and promotes the repair of DSBs occurring within different heterochromatin compartments. DSB repair pathways operating in each case are indicated.

A role for the nucleoskeleton and molecular motors in the relocalization of heterochromatic breaks was put forward in several species (Figure 2). In *Drosophila* for instance, nuclear actin filaments and myosins promote the relocalization of pericentromeric DSBs to the nuclear periphery (Caridi et al., 2018; Dialynas et al., 2019), with a contribution of sumoylation and of structural maintenance of chromosomes 5/6 proteins (SMC5/6) in anchoring the breaks to the nuclear periphery (Ryu et al., 2015). Mechanisms appear to be distinct in mammalian cells where SMC5/6 proteins are dispensable, and anchoring of breaks to the nuclear periphery does not occur (Tsouroula et al., 2016). Nuclear actin drives the migration of a subset of breaks undergoing HR also in mammalian cells (Schrank et al., 2018), but whether those correspond to heterochromatic breaks is not known. Similar to DSBs in pericentromeric heterochromatic repeats, DSBs in nucleolar repeats trigger chromatin mobility. Indeed, in human cells, nucleolar DSBs relocalize to the periphery of nucleoli where they are repaired by HR (van Sluis and McStay, 2015). A recent study provided the first clue to molecular players controlling the relocalization of nucleolar breaks, with a role for myosin chaperones and actin-related proteins (Marnef et al., 2019). The linker of the nucleoskeleton and cytoskeleton (LINC) complex, embedded in the nuclear envelope, also contributes to nucleolar DSB mobility, which involves nuclear envelope invaginations that connect nucleoli (Marnef et al., 2019). Similarly, DSB repair by NHEJ in heterochromatin domains invokes microtubule-mediated chromatin mobility, as reported for the fusion of uncapped telomeres in mouse cells, which is promoted by 53BP1-dependent chromatin mobility through the LINC complex (Dimitrova et al., 2008; Lottersberger et al., 2015). This microtubule-mediated heterochromatin mobility stimulates NHEJ of dysfunctional telomeres (Lottersberger et al., 2015). Together, these studies put forward the role of the nucleoskeleton in regulating damaged heterochromatin mobility to support DSB repair in several contexts (Figure 2). Considering the emerging role of phase separation in regulating DNA damage responses (Spegg and Altmeyer, 2021), we can envision molecular condensates as part of an alternative or cooperative mechanism to control DSB dynamics.

### Maintenance of Heterochromatin Organization in Response to DSBs

Heterochromatin regions play a crucial role in silencing transposable elements and in regulating the segregation and stability of chromosomes (Allshire and Madhani, 2018). Thus, the maintenance of heterochromatin features is essential to preserve genome integrity and cell identity. However, very little is known regarding whether and how heterochromatin organization is faithfully re-established after DSB repair. One would assume that the heterochromatin compaction state would be restored and that repaired loci would retrieve their original positions inside heterochromatin domains. To address these questions, long-term kinetic analyses should be carried out post DSB induction in order to follow changes in heterochromatin organization during the course of the repair process and even beyond DSB repair completion. A refined analysis of heterochromatin folding during DSB repair would also be needed. Several recent studies have shed light on the impact of DSBs on chromatin folding in the nuclear space and on the importance of 3D chromatin organization in shaping DSB responses by exploiting chromatin conformation capture and super-resolution microscopy (Natale et al., 2017; Ochs et al., 2019; Sanders et al., 2020; Arnould et al., 2021). Similar approaches would help to determine whether heterochromatin compartments retrieve their original topology after DSB repair and to dissect the underlying molecular mechanisms.

Despite dramatic changes in heterochromatin organization following DNA breaks, some heterochromatin marks are maintained during the DSB repair process, as shown for H3K9me3 in mouse pericentromeric heterochromatin (Tsouroula et al., 2016; Natale et al., 2017) but not in *Drosophila*, where H3K9me3 levels decrease post DSB (Janssen et al., 2019). The mechanisms supporting the maintenance or the restoration of H3K9me3 within these heterochromatic domains are not yet elucidated; however, it is tempting to envision a similar response to what is observed in UV-damaged pericentromeric heterochromatin domains in mouse cells, where the histone methyltransferase SETDB1 is recruited and coordinates the maintenance of H3K9me3 with new H3 deposition during UV damage repair (Fortuny et al., 2021).

### Removal of Heterochromatin Features From Euchromatin Domains Following DSB Repair

**Figure 3:**
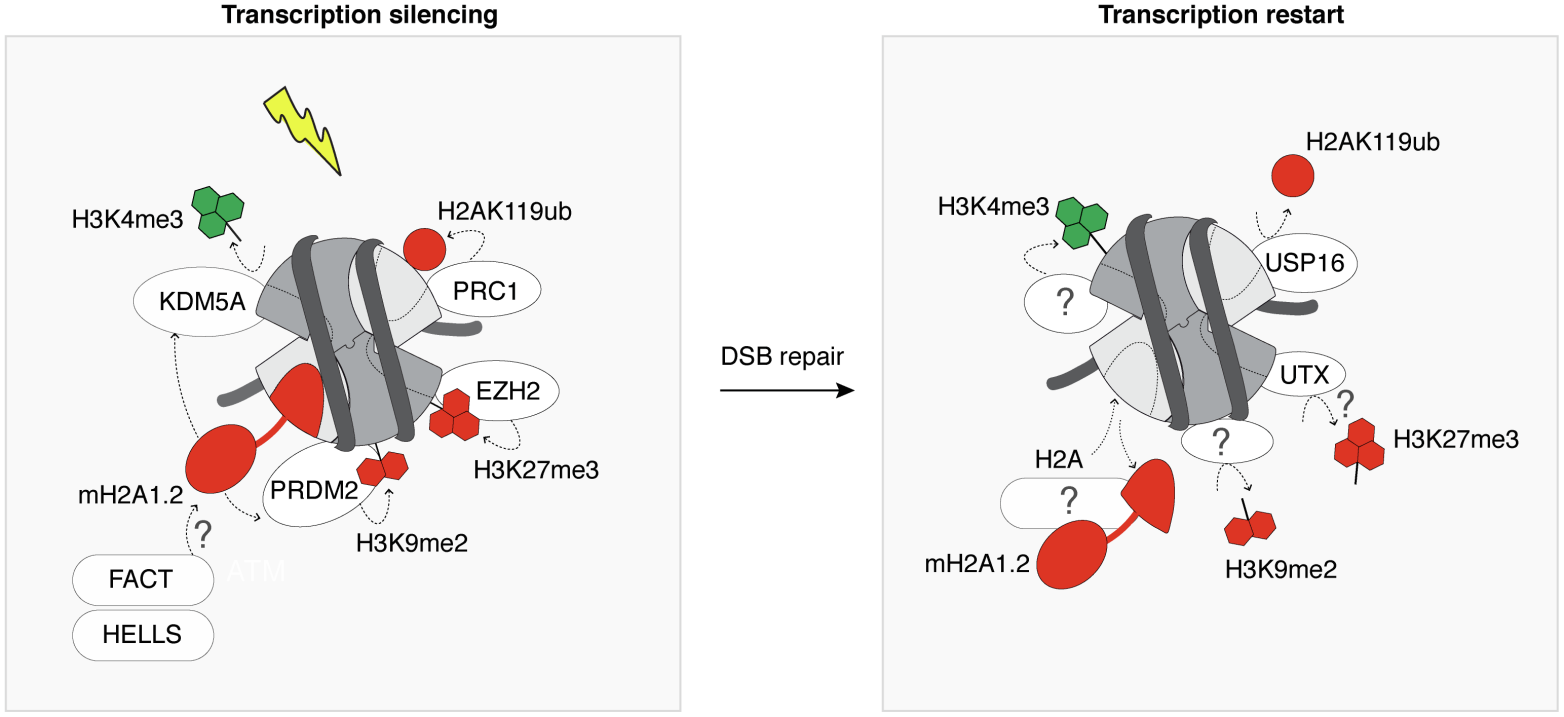
Heterochromatin features are transiently established following DSBs. Following DSB induction, the chromatin surrounding the lesion is modified by the deposition of heterochromatin-specific histone variants and post-translational modifications (red) at the expense of active marks (green). This contributes to silence transcription in the vicinity of the DSB. For simplicity, all chromatin marks are represented on a single nucleosome flanking a DSB. Most of the players that promote the recovery of the pre-existing marks after completion of DSB repair are still unknown, as indicated by the question marks. White ovals and rectangles represent histone modifying enzymes and histone chaperone/remodeler, respectively.

DNA double-strand breaks within transcribed genes trigger the incorporation of heterochromatin-specific histone variants and histone post-translational modifications, leading to a transient heterochromatinization of the damaged locus, which contributes to transcriptional silencing (Figure 3). Among the many regulators of transcriptional silencing at DSBs (Caron et al., 2019), heterochromatin marks play a pivotal role. For instance, mono-ubiquitylation of H2A on Lys 119 is induced in the vicinity of DSBs and governs transcriptional silencing (Shanbhag et al., 2010). Interestingly, the removal of H2AK119ub involves the deubiquitinase ubiquitin-specific peptidase 16 (USP16) and is crucial for transcription restart after DSB repair (Shanbhag et al., 2010). Silencing at DSBs is also contributed to by the deposition of H3K27me3 through the PARP1-EZH2 axis (Abu-Zhayia et al., 2018). However, how this histone mark is removed once the break is repaired and whether it is required to license transcription restart is still unknown. The histone demethylase ubiquitously-transcribed tetratricopeptide repeat X (UTX) was shown to diminish H3K27me3 levels in response to ionizing radiation (Rath et al., 2018). It is thus tempting to speculate that the removal of H3K27me3 upon DSB repair may be mediated by UTX, contributing to transcription restart. The transient heterochromatinization at euchromatic breaks also involves H3K9me2/3 deposition (Ayrapetov et al., 2014; Khurana et al., 2014). H3K9 dimethylation is deposited by PR/SET domain 2 (PRDM2), recruited to DSBs in a manner dependent on the histone variant macroH2A1.2 (Khurana et al., 2014). This histone variant also inhibits transcription at DSBs by stimulating H3K4me3 demethylation by KDM5A (Kumbhar et al., 2021). Further studies will be needed to investigate reversal mechanisms of these heterochromatin marks after DSB repair, including the removal of macroH2A1.2, H3K9me2/3, and their importance for transcription recovery.

## Conclusion and Future Directions

During the last decade, a growing number of studies have highlighted the key contribution of histone post-translational modifications and factors implicated in heterochromatin formation in the response to DSBs. Heterochromatin marks, either pre-existing in heterochromatin domains or *de novo* established in euchromatin, indeed play a central role in regulating DSB repair pathway choice. Thus, heterochromatin features should not be considered as barriers to DSB repair but as fine-tuners of the DSB response. While our knowledge of the DSB repair pathways that operate in different heterochromatin domains is increasing, the players involved in restoring heterochromatin organization and in erasing heterochromatin marks from euchromatin regions after DSB repair are still unknown. Beyond histone variants and modifications, another crucial epigenetic mark enriched in heterochromatin domains is DNA methylation. Interestingly, DSB repair alters DNA methylation patterns (Sriraman et al., 2020), but little is known about the mechanisms allowing DNA methylation restoration. Furthermore, the DNA methyltransferase DNMT1 can read heterochromatin histone marks thus protecting cells against ionizing radiation (Ren et al., 2020, 2021). These findings suggest a potential role for DNA methylation in controlling DSB repair responses, which is still to be elucidated. Future work will shed light on these mechanisms and on the interplay between different heterochromatin marks in regulating DSB responses. This will help move toward a better characterization of genome and epigenome maintenance processes whose defects underlie pathological disorders.

## Author Contributions

All authors contributed to write this review and approved the submitted version.

## Conflict of Interest

The authors declare that the research was conducted in the absence of any commercial or financial relationships that could be construed as a potential conflict of interest.

## Publisher’s Note

All claims expressed in this article are solely those of the authors and do not necessarily represent those of their affiliated organizations, or those of the publisher, the editors and the reviewers. Any product that may be evaluated in this article, or claim that may be made by its manufacturer, is not guaranteed or endorsed by the publisher.
